# Mortuary Statistics

**Published:** 1881-01

**Authors:** 


### NOVEM'R REPORT OF DEATHS AND INTERMENTS IN BALTIMORE.

| CAUSE OF DEATH. | No. | CAUSE OF DEATH. | No. | CAUSE OF DEATH. | No. |
|---|---|---|---|---|---|
| Abscess | 2 | Dropsy, (Gen'l) | 3 | Marasmus | 16 |
| Apoplexy | 7 | " Heart | 1 | Meningitis | 6 |
| Asphyxia | 1 | " Scarlatinal | 1 | " Tubercular | 7 |
| Asthma | 1 | Drowned | 1 | " Cerebro Spinal | 1 |
| Asthenia | 5 | Dysentery | 1 | Neuralgia | 1 |
| Bright's Disease | 8 | Emphysema | 1 | Old Age | 21 |
| Burns | 5 | Epilepsy | 1 | Paralysis | 15 |
| Capillary Bronchitis.' | 6 | Erysipelas | 2 | Pneumonia | 35 |
| Cancer, Abdomen | 1 | Embolism | 2 | " Typhoid | 2 |
| " Breast | 2 | Exposure | 1 | " Broncho | 1 |
| " Leg | 1 | Fever, Catarrhal | 3 | " Pleuro | 1 |
| " Liver | 2 | " Gastric | 1 | Poison | 1 |
| " Face | 1 | " Malarial | 3 | Premature Birth | 13 |
| " Head | 1 | " Puerperal | 3 | Protracted Labor | 1 |
| " Mediastrinal Space | 1 | " Remittent | 1 | Phthisis Laryngeal. | 1 |
| " Intestines | 1 | " Scarlet | 33 | Pyæmia | 1 |
| " Uterus | 1 | " Typhoid | 21 | Rheumatism | 4 |
| " Stomach | 3 | " Typho-Malarial | 5 | " Heart | 1 |
| Child Birth | 2 | Fracture, Thigh | 1 | Stomatitis | 1 |
| Cholera Infantum | 2 | " Skull | 2 | Softening of Brain | 2 |
| Congestion of Brain | 6 | Gout | 1 | Scalds | 1 |
| " Lungs | 2 | Hernia, Strangulated | 1 | Spina Bafida | 1 |
| Consumption of Bowels | 2 | Hemorrhage, Brain | 2 | Syphilis | 2 |
| " Lungs | 94 | Hydrocephalus | 3 | Tabes Mesenterica | 1 |
| Convulsions | 27 | Inanition | 9 | Thrombosis | 1 |
| Catarrh | 3 | Inflammation of Brain | 1 | Trismus Nascentium | 2 |
| Croup | 24 | " Bowels | 3 | Tumor above origin of the Pnemogastric Nerve | 1 |
| Congestive Chills | 1 | " Stomach | 7 | Tumor, Brain | 1 |
| Cyanosis | 1 | " Bronchi | 6 | Ulcer, Uterine | 1 |
| Colic | 1 | " Kidneys | 2 | Uræmia | 1 |
| Dentition | 4 | " Larynx | 3 | Unknown, Adult | 1 |
| Dyspepsia | 2 | " Pericard'm | 1 | Unknown, Infantile | 4 |
| Diarrhœa | 4 | " Peritoneum | 6 | Whooping Cough | 9 |
| Diphtheria | 23 | " Pleura | 1 | Wounds | 4 |
| Disease of Hip Joint | 1 | Intemperance | 2 | | |
| " Heart | 15 | Malformation | 1 | Total | 563 |
| " Stomach | 1 | Mal-nutrition | 1 | | |
| " Spine | 1 | | | | |

THE DEATHS REGISTERED IN THE MONTH INCLUDE

| | | | |
|---|---|---|---|
| Under 1 year | 130 | Between 20 and 30 " | 50 |
| Between 1 and 2 years | 35 | " 30 and 40 " | 46 |
| " 2 and 5 years | 43 | " 40 and 50 " | 39 |
| | | " 50 and 60 " | 38 |
| Under 5 years | 208 | " 60 and 70 " | 37 |
| Between 5 and 10 years | 53 | " 70 and 80 " | 38 |
| " 10 and 15 " | 18 | " 80 and 90 " | 17 |
| " 15 and 20 " | 17 | " 90 and 100 " | 2 |

NATIVITY, ETC.

| | |
|---|---|
| United States—White | 337 |
| " Colored | 129 |
| Foreign | 97 |
| Still Birth | 61 |
| Total for the month | 563 |

Among the decedents were 58 above the age of 70 years, whose combined ages were 4,531 years, averaging 78 years 1 month.

Estimated Population, 393,796. White, 338,384. Colored, 55,412. Annual Death Rate during the month, whole population, per 1,000—17.15. White, 15.40. Colored, 28.14.

By Order: JAMES A. STEUART, M. D., Commissioner of Health and Registrar.

A. R. CARTER, Secretary.

U. S. SIGNAL OFFICE, BALTIMORE, MD., December 1, 1880.

### METEOROLOGICAL SUMMARY FOR THE MONTH OF NOVEMBER, 1880.

### PRESSURE.

| | | |
|---|---|---|
| Mean Monthly Barometer, | 30.270 inches. | |
| " " " for past 10 years, | 30.110 " | |
| Highest Barometer, | 30.795 " | on the 24th. |
| Lowest " | 29.496 " | on the 6th. |
| Monthly Range of Barometer, | 1.299 " | |

### TEMPERATURE.

Mean Monthly Thermometer, 42.1 degrees.
" " " for past 10 years, 44.9 "
Highest Thermometer, 69 degrees on 5th. Monthly Range, 54 degrees.
Lowest " 15 " on 22d. Mean Daily Range, 12.8 "

### WIND, ETC.

Prevailing Direction of Wind, Northwast.
MeanVelocity of Wind, 5.5 miles per hour.
Highest Velocity of Wind, 26 " " on 7th.
Total Movement of Wind, 3,640 miles.

Total Amount of Rainfall, 2.86 inches.
Number of Days on which Rain fell, 15.
Mean Relative Humidity, 66.1 per cent.

ROBERT SEYBOTH, in charge.

